# Reproducibility of Densitometric and Biomechanical Assessment of the Mouse Tibia From *In Vivo* Micro-CT Images

**DOI:** 10.3389/fendo.2022.915938

**Published:** 2022-06-30

**Authors:** Sara Oliviero, Vee San Cheong, Bryant C. Roberts, Carlos Amnael Orozco Diaz, William Griffiths, Ilaria Bellantuono, Enrico Dall’Ara

**Affiliations:** ^1^ Department of Oncology and Metabolism, Mellanby Centre for bone Research, University of Sheffield, Sheffield, United Kingdom; ^2^ INSIGNEO Institute for In Silico Medicine, University of Sheffield, Sheffield, United Kingdom; ^3^ Department of Industrial Engineering, Alma Mater Studiorum, University of Bologna, Bologna, Italy; ^4^ Medical Technology Lab, IRCCS Istituto Ortopedico Rizzoli, Bologna, Italy; ^5^ Department of Automatic Control and Systems Engineering, University of Sheffield, Sheffield, United Kingdom; ^6^ Healthy Lifespan Institute, University of Sheffield, Sheffield, United Kingdom

**Keywords:** reproducibility, mouse tibia, microCT, morphometric, bone mineral, finite element

## Abstract

Interventions for bone diseases (e.g. osteoporosis) require testing in animal models before clinical translation and the mouse tibia is among the most common tested anatomical sites. *In vivo* micro-Computed Tomography (microCT) based measurements of the geometrical and densitometric properties are non-invasive and therefore constitute an important tool in preclinical studies. Moreover, validated micro-Finite Element (microFE) models can be used for predicting the bone mechanical properties non-invasively. However, considering that the image processing pipeline requires operator-dependant steps, the reproducibility of these measurements has to be assessed. The aim of this study was to evaluate the intra- and inter-operator reproducibility of several bone parameters measured from microCT images. Ten *in vivo* microCT images of the right tibia of five mice (at 18 and 22 weeks of age) were processed. One experienced operator (intra-operator analysis) and three different operators (inter-operator) aligned each image to a reference through a rigid registration and selected a volume of interest below the growth plate. From each image the following parameters were measured: total bone mineral content (BMC) and density (BMD), BMC in 40 subregions (ten longitudinal sections, four quadrants), microFE-based stiffness and failure load. Intra-operator reproducibility was acceptable for all parameters (precision error, PE < 3.71%), with lowest reproducibility for stiffness (3.06% at week 18, 3.71% at week 22). The inter-operator reproducibility was slightly lower (PE < 4.25%), although still acceptable for assessing the properties of most interventions. The lowest reproducibility was found for BMC in the lateral sector at the midshaft (PE = 4.25%). Densitometric parameters were more reproducible than most standard morphometric parameters calculated in the proximal trabecular bone. In conclusion, microCT and microFE models provide reproducible measurements for non-invasive assessment of the mouse tibia properties.

## Introduction

Osteoporosis and osteoarthritis are among the most common chronic diseases of the musculoskeletal system. Animal models are fundamental for the development and testing of new bone biomechanical or pharmacological interventions before clinical translation, and the mouse is the most common animal model. Its advantages include the ability to control the animal environment, the relatively low costs, and the possibility to perform high-resolution imaging of bone and other musculoskeletal tissues (1). In particular, the ability to perform micro-Computed Tomography (microCT) imaging *in vivo* in a longitudinal experimental study (2) improves measurement accuracy by reducing the inter-subject variability due to a cross-sectional design. Additionally, this approach can dramatically reduce the usage of mice in bone research, in line with the 3Rs (replacement, refinement and reduction of the usage of animals in research) (3). In terms of clinical translation, bone densitometric and mechanical properties are relevant endpoints in animal studies, since similar parameters are measured in patients in the clinical practice or in clinical research. Bone mineral density (BMD) and bone strength are strongly associated and therefore major predictors of fracture risk (4–6). MicroCT imaging of the mouse tibia is extensively used to measure morphometric parameters in the cortical and trabecular compartments. A method has been previously proposed to assess the spatial distribution of bone mineral content (BMC) and BMD over the whole tibia volume (7), by dividing the tibia into 10 longitudinal sections (from proximal to distal) and 4 quadrants (anterior, posterior, medial and lateral), giving a total of 40 partitions. Micro-Finite Element (microFE) models based on microCT data can be used for predicting the bone mechanical response under compression non-invasively. These models have been recently validated against experimental tests, with errors associated to the predictions of bone strength of 9% (8) and good prediction of local deformation (9). The microCT-based parameters have been applied to study the effect of different bone interventions, including ovariectomy (10), mechanical loading (11–14) and parathyroid hormone injections (PTH) (15). Moreover, the microFE models have been used to predict bone apposition over time and space due to bone anabolic treatments, such as mechanical loading (16) and/or injections of PTH (17).

Nevertheless, the reproducibility of microCT-based measurements is affected by different factors associated with the operator-dependant evaluation of the images, e.g. threshold value used for segmentation, image alignment, selection of region of interest. Kohler et al. (18) investigated the intra-operator reproducibility of trabecular and cortical parameters measured in the mouse femur of two different strains (C3H and SJL), by acquiring five repeated scans. Precision errors were in the range of 0.59% to 5.24%, with lowest reproducibility found for connectivity density. Verdelis and colleagues (19) analysed the reproducibility of microCT measurements obtained from three different microCT systems in the trabecular bone of the distal femur of C57BL/6 mice, by performing three scans without repositioning for each system. Coefficients of variation were lower than 5% intra-system, while inter-system differences of up to 236% for trabecular thickness were found. These large differences could be due to the systematic differences in assessing some morphometric properties of bone with different software packages (20). Christiansen (21) analysed the effect of voxel size (from 6 to 30 µm) and segmentation method (qualitatively selected by the operator vs quantitatively selected based on the image histogram) on trabecular morphometric parameters measured in the L5 vertebra of C57BL/6N mice. Differences of up to 126% for trabecular thickness and 44% for tissue BMD were obtained when scanning at different voxel size. Differences between the two threshold methods were between 1% and 8% for voxel sizes between 6µm and 10µm. Nishiyama et al. (22) reported precision errors of lower than 8.40% for trabecular and cortical morphometric parameters (connectivity density), obtained from four repeated scans for two mouse strains (C57BL/6J and C3H/HeJ). Lu and colleagues (7) performed four repeated scans of C57BL/6J mouse tibiae *in vivo* and evaluated the reproducibility of the BMC calculated in 40 partitions (ten longitudinal sections, four sectors). Precision errors were lower than 3.5% for all partitions. In (23), the effect of the longitudinal alignment of the C57Bl/6J mouse tibia in FE models was evaluated by varying the orientation of the proximal-distal axis in the range of 0° to 20°. Local strains magnitude obtained by simulating longitudinal compression varied up to 40% due to tibia misalignments. In a recent work, Gardegaront et al. analysed the inter-operator (two operators) reproducibility of failure load estimated from microCT-based finite element models of BALB/C mouse tibiae with induced bone tumor or sham, which aimed to replicate the experimental longitudinal compression (24). They found differences between 9.8% and 58.3% depending on the method used to implement boundary conditions. Additionally, by varying the orientation of the tibia in the range of -5° to +5° in the sagittal and coronal planes, differences were in the range of 9.1% to 44.7%.

According to the results reported in (7), measurements of the spatial distribution of BMC in the tibia have a high reproducibility, generally better than standard morphometric parameters. However, their intra- and inter-operator reproducibility has never been reported. In particular, the image alignment and selection of volume of interest rely on operator-dependant tasks, which may add uncertainty to the outcomes, despite the definition of a protocol followed by the operators. Similarly (23), and (24) demonstrated that the tibia alignment may have an influence on the mechanical properties predicted by microFE models.

The aim of this study was to quantify the intra- and inter-operator reproducibility of densitometric and mechanical properties estimated from *in vivo* microCT images and validated microFE models of the mouse tibia.

## Materials and Methods

**Figure 1:**
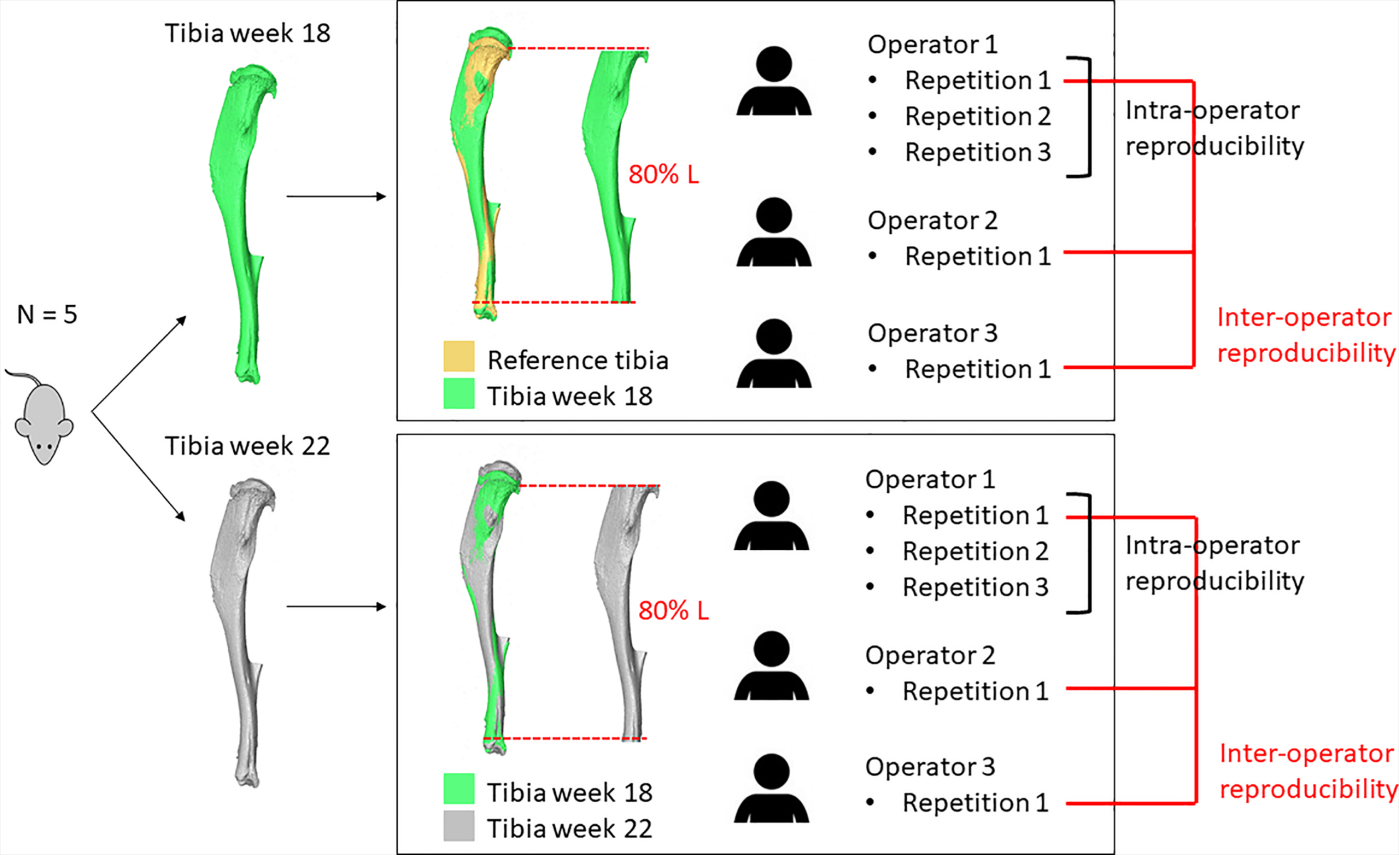
Overview of the study. The right tibia of five mice was microCT scanned longitudinally at week 18 and week 22 of age. Each image acquired at week 18 of age was aligned to a reference and a volume of interest (VOI, 80% Length) was selected. Each image acquired at week 22 of age was aligned to the corresponding baseline image and a VOI was selected. These operations were repeated three times by operator 1 and once by operators 2 and 3.

An overview of the methods used in this study is presented in **Figure 1**. Briefly, ten microCT images of the right tibiae of five mice acquired in a previous study (13) were used to assess the reproducibility of different microCT based parameters. Five images were acquired at 18 weeks of age and five at 22 weeks of age longitudinally for the same mice. Each tibia was registered to a reference in order to align them in the same spatial orientation. A volume of interest was selected for BMC, BMD and microFE analyses. For intra-operator reproducibility, one operator repeated these tasks three times. For inter-operator reproducibility, two additional operators repeated the tasks once. From each aligned image, the densitometric and mechanical properties were evaluated and compared.

### Scanning Procedures and Reconstructions

The right tibiae of six C57BL/6J female mice were microCT scanned *in vivo* in a previous study between the age of 14 and 24 weeks, every two weeks (13). Mice were ovariectomized at the age of 14 weeks to simulate postmenopausal osteoporosis. At the age of 18 weeks, mice were treated with PTH injections for 5 days/week until the age of 22. The microCT images acquired at the age of 18 weeks (treatment onset) and 22 weeks (treatment withdrawal) were used in this study. The applied scanning procedure has been previously optimized for *in vivo* applications (VivaCT 80, Scanco Medical, Bruettisellen, Switzerland; 55 kVp, 145 μA, 10.4 μm voxel size, 100 ms integration time, 32 mm field of view, 750 projections/180°, no frame averaging, 0.5 mm Al filter) as a compromise between nominal radiation dose and accuracy in the measurement of bone properties (25). This protocol requires 25 minutes (5 scan rotations) to scan the whole tibia, and is associated to a nominal radiation dose of 256 mGy, which has minimal effects on the bone properties (26). All images were reconstructed using the software provided by the manufacturer (Scanco Medical AG) and applying a beam hardening correction based on a wedge phantom with 1200 mg HA/cc density, which has been shown to improve the local tissue mineralization measurement (27).

### Image Registration and Volume of Interest

One of the six images acquired at week 18 of age was used as reference and aligned so that the proximal-distal axis defined according to (12) was aligned to the *z* direction of the global reference system. Each of the five remaining images was rigidly registered to the reference (Amira 6.0.0, FEI Visualization Sciences Group, France) using Normalized Mutual Information as the optimization criterion. Images were resampled using Lanczos interpolator (28). Each image acquired at week 22 was rigidly registered to the corresponding baseline image (scan at week 18). After alignment, a Gaussian filter (kernel 3x3x3, standard deviation 0.65) was applied to reduce the high frequency noise (1). A volume of interest (VOI) was selected below the growth plate, starting from the cross-section where the growth plate tissue was not visible anymore. In order to take into account for the small increase of tibia length between week 18 and 22 of age, the VOI included 80% of the total length (L) of the tibia and excluded the fibula. The fibula was excluded by applying a 2D connectivity filter at the cross-sections around the tibio-fibular junction, followed by a 3D connectivity filter applied on the whole tibia.

### Standard Morphometric Analysis

The intra-operator reproducibility for standard morphometric measurements in the trabecular and cortical bone of the mouse tibia (1) was evaluated for comparison with other methods. The procedure applied for morphometric analysis has been published previously (25, 26) and is briefly summarized here. Standard morphometric analyses of trabecular and cortical regions of interest were performed in CTAn (Bruker, Belgium). For trabecular analysis, to take into account for possible differences in proximal features within each group and for increased tibia length between week 18 and week 22 of age, a reference cross-section was selected, identified as the one where the medial and lateral sides of the growth plate merged. The trabecular VOI started at an offset of 0.2 mm from the reference slice and extended 1 mm distally. Trabecular bone was contoured manually by selecting 2D regions of interest every 5 slices. A single level threshold was used for segmentation, calculated as the average of threshold values for each tibia, as chosen by the operator by comparing the greyscale and binary datasets. A despeckling filter was applied to remove 3D white (bone) regions less than 10 voxels in volume. Trabecular bone volume fraction (Tb.BV/TV, [%]), thickness (Tb.Th, [µm]), separation (Tb.Sp, [µm]), number (Tb.N, [1/mm]), connectivity density with assumption of connectivity around the boundary (Conn.D, [1/mm^3^]) and degree of anisotropy (DA, [-]) were computed (1).

For cortical analysis, a 1 mm thick region was centered at the tibial midshaft. After segmentation, pores within the cortex were removed by applying a closing function (2D round kernel, 10 pixels radius). Total cross-sectional area (Tt.Ar, [mm^2^]), cortical bone area (Ct.Ar, [mm^2^]), cortical area fraction (Ct.Ar/Tt.Ar, [mm^2^/mm^2^]) and cortical thickness (Ct.Th, [µm]) were computed (1).

### Spatial Distribution of BMC

The method used for analyzing the spatial distribution of bone mineral content has been reported in (7, 25) and is summarized here. The attenuation coefficients acquired in the microCT images were converted into tissue mineral density (TMD, [mgHA/cc]) by using the calibration law provided by the manufacturer of the scanner. Weekly quality checks were performed on a densitometric phantom with five insertions (800, 400, 200, 100 and 0 mgHA/cc) in order to monitor the stability of the calibration parameters. BMC in each voxel was calculated as its TMD multiplied by the volume of the voxel. For the VOI (80% of tibia length) the following parameters were obtained: total bone mineral content (BMC, [mg]), total bone mineral density (BMD, [mg HA/cc]), total tissue mineral density (TMD, [mg HA/cc]), total bone volume fraction (BV/TV, [%]), average and minimum cross-sectional area (TotArea, [mm^2^]), average and minimum bone area (BoneArea, [mm^2^]). Subsequently, the VOI was divided into ten longitudinal sections (from 1 at the proximal end to 10 at the distal end) and each longitudinal section was divided into four quadrants (anterior, posterior, medial and lateral), defined for each cross-section by two perpendicular lines containing its centroid (40 partitions in total). BMC was calculated in each partition.

### Micro-Finite Element Models

After selecting the VOI for each tibia, each image was segmented by using a specimen-specific global threshold, calculated as the average of the grey levels corresponding to the bone and background peaks in the image histogram (21). A connectivity filter was applied to remove unconnected voxels (connectivity rule equal to 6, keeping plane connectivity, bwlabeln function in Matlab). A Cartesian mesh was obtained by converting each bone voxel into an 8-noded hexahedral element (11, 29) with isotropic linear elastic material properties [Young’s Modulus = 14.8 GPa, Poisson’s ratio = 0.3 (9)]. Uniaxial compression was simulated by fully constraining the distal end of the tibia and applying a displacement of 0.1 mm on each node of the proximal surface along the longitudinal direction (Ansys, Release 15.0, ANSYS, Inc.). The apparent stiffness ([N/mm]) was calculated as the sum of reaction forces at the distal surface, divided by the applied displacement. The failure load ([N]) was estimated from linear microFE models using a failure criterion optimized for the mouse tibia (8), that assumes that the bone fails when 10% of the nodes reach third principal strain values equal to -14420 µϵ.

### Intra- and Inter-Operator Analysis

The two steps described in section 2.2 (image registration and VOI selection) were the operator-dependent tasks of the pipeline. The image registration is affected by the initial position selected by the operator before initializing the automatic registration algorithm. Additionally, the operator may have to manually adjust the tibia orientation in case of a residual misalignment. The VOI selection is performed by selecting the first cross-section of the VOI, defined as the cross-section where the growth plate tissue is not visible anymore. Subsequently, the selection of the VOI is another potential source of error. Afterwards, the tibia length is measured by finding the slices corresponding to the most proximal and most distal voxels, and 80% of the total length is selected. Guidelines were created and agreed upon by all three operators for performing these steps. For intra-operator analysis, one experienced operator repeated these steps three times, while for inter-operator analysis, two additional operators performed them once.

### Description of the Webservice

**Figure 2:**
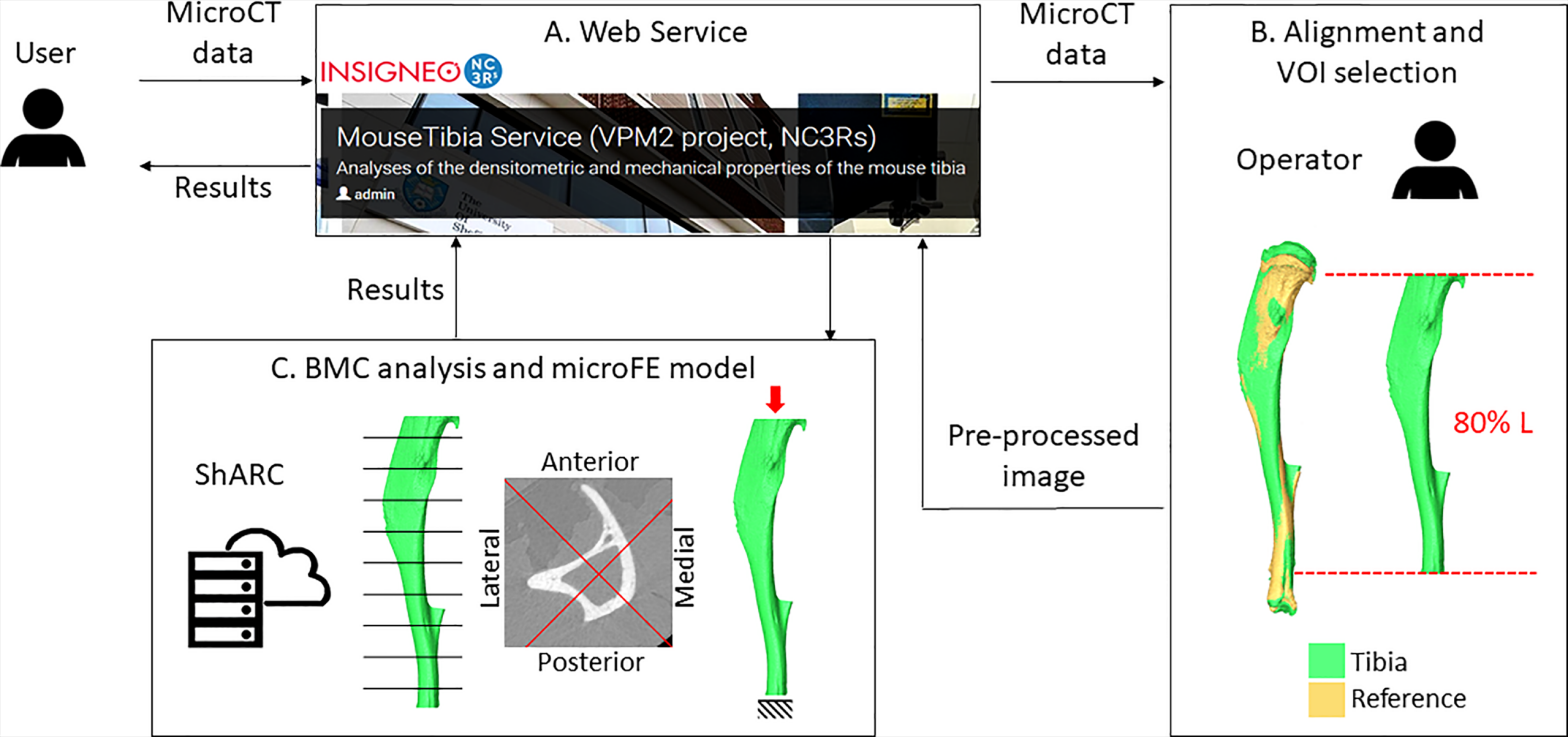
Overview of the workflow. A user uploads microCT data to the webservice **(A)**. An operator downloads the image and performs the alignment and VOI selection **(B)**. Pre-processed images are uploaded back to the webservice. Bone mineral content (BMC) and microFE analyses **(C)** are run automatically in the HPC ShARC (Sheffield Advanced Research Computer). The results are sent back to the service and to the user.

A webservice (https://mousetibia.insigneo.org/uct2ufe/) based on the analyses described in 2.4 and 2.5 (7, 8, 30) has been developed, where users can upload microCT images of the mouse tibia and run the BMC and microFE analyses (**Figure 2**). The service requires an operator to pre-process the uploaded images as described (section 2.2). All subsequent steps in the pipeline (section 2.4-2.5) are fully automatic and run on a high-performance computing (HPC) server (ShARC, Sheffield Advanced Research Computer, University of Sheffield). A more detailed schematic of the webservice can be found in the **Supplementary Material** (**Supplementary Figure 1**). Briefly, the service relies on a web application, two Ubuntu daemons and a Python/Matlab/Ansys HPC script. These applications message each other using RabbitMQ and transfer DICOM data to each other using the Google Drive API. Two workflows are triggered by the user and operator respectively. The first workflow is triggered when a user submits job data and microCT data, while the second is triggered by the operator when they have completed image pre-processing (**Supplementary Figure 1**). This automatically runs the code for BMC and FE analyses, and returns to the web application and the user the results of the analyses in a report containing the densitometric and mechanical properties calculated for the tibia.

In this study each operator used the webservice for BMC and microFE analyses.

### Statistical Analysis

Three metrics were used to assess the reproducibility of each parameter.

Precision error (PE) was calculated as follows:


$$PE=\sqrt{\sum_{j=1}^{m}\frac{CV_{j}^{2}}{m}};$$

$$CV=\frac{SD}{\text{μ}}$$

here CV is the coefficient of variation, calculated as the standard deviation (SD) of measurements divided by the average (µ), *m* is the number of subjects (equal to 5 in this study).

Least significant change (LSC) is defined as


$$LSC=PE\;Z\sqrt{\frac{1}{n_{1}}+\frac{1}{n_{2}}}$$

where *Z* is the *Z* score for a two tailed 95% confidence level (Z = 1.96), *n*_1_ is the number of measures at baseline (equal to 1 in this study), *n*_2_ is the number of measures at follow up time points (equal to 2 in this study).

Intraclass correlation coefficient (ICC) was obtained using SPSS (Reliability analysis, IBM SPSS Statistics 25) by using the following settings: two-way random model, absolute agreement, single measures.

The effect of PTH was calculated for each mouse as the difference between each parameter measured at week 22 and at week 18 of age, normalized by the measurement at week 18 of age. Afterwards, the average and standard deviation for the five mice were calculated for each repetition by the same operator or by different operators.

## Results

Reproducibility of morphometric parameters is presented in **Table 1**. The lowest reproducibility was found for connectivity density (PE = 8.66% at week 18, PE = 8.87% at week 22) and trabecular bone volume fraction (PE = 5.85% at week 18). The PEs for other parameters in the trabecular bone were lower than 5%. As expected, cortical parameters were more reproducible than trabecular parameters, with PE below 1% in all cases.

**Table 1:** Intra-operator reproducibility of morphometric parameters.

	PE [%]		LSC [%]		ICC [-]	
	Week 18	Week 22	Week 18	Week 22	Week 18	Week 22
Trabecular morphometric parameters						
Tb.BV/TV	5.85	3.75	14.04	9.01	0.832	0.972
Tb.Th	1.30	0.91	3.11	2.19	0.930	0.992
Tb.Sp	2.90	2.37	6.96	5.68	0.825	0.976
Tb.N	4.87	3.83	11.70	9.18	0.878	0.956
Conn.D	8.66	8.87	20.78	21.29	0.935	0.909
DA	2.52	0.98	6.06	2.34	0.780	0.993
Cortical morphometric parameters						
Tt.Ar	0.08	0.14	0.18	0.33	0.999	0.998
Ct.Ar	0.08	0.12	0.18	0.29	0.999	0.999
Ct.Ar/Tt.Ar	0.02	0.03	0.04	0.08	1.000	1.000
Ct.Th	0.16	0.29	0.39	0.69	0.990	0.995

PE, precision error; LSC, least significant change; ICC, intraclass correlation coefficient.

The statistical analyses for intra-operator and inter-operator reproducibility of measurements of densitometric and mechanical parameters are reported in **Tables 2** and **3**, respectively. PEs for BMC and mechanical properties were generally lower compared to standard morphometric parameters, both for intra-operator and inter-operator assessments. The lowest reproducibility was found for structural stiffness estimated with FE models (PE=3.71% intra-operator, 4.09% inter-operator), and for BMC in one of the 40 partitions at week 22 (section 4 lateral, PE=4.25% inter-operator). The PE for predicted bone strength was lower than most morphometric parameters. Inter-operator reproducibility was lower than intra-operator.

**Table 2:** Intra-operator reproducibility of densitometric and mechanical parameters.

	PE [%]		LSC [%]		ICC [-]	
	Week 18	Week 22	Week 18	Week 22	Week 18	Week 22
L	0.03	0.04	0.07	0.09	1.000	1.000
Tot BMC	0.05	0.05	0.11	0.13	1.000	1.000
Tot TMD	0.07	0.09	0.17	0.23	0.992	0.992
Tot BV	0.11	0.15	0.27	0.35	1.000	0.999
Tot TV	0.17	0.17	0.40	0.40	0.999	0.999
Tot BMD	0.15	0.13	0.36	0.31	0.998	0.999
Tot BV/TV	0.12	0.10	0.30	0.23	0.998	0.999
AvTotArea	0.15	0.14	0.37	0.34	0.998	0.999
AvBoneArea	0.11	0.12	0.26	0.28	0.999	0.999
MinTotArea	0.08	0.07	0.20	0.18	0.999	0.999
MinBoneArea	0.20	0.13	0.48	0.32	0.998	0.999
BMC in 40 sectors	0.15 –2.01	0.14 –2.38	0.36 – 4.83	0.34 – 5.72	0.824 – 1.000	0.703 – 1.000
Stiffness	3.06	3.71	7.36	8.92	0.838	0.758
Failure load	1.51	1.80	3.62	4.33	0.905	0.879

PE, precision error; LSC, least significant change; ICC, intraclass correlation coefficient.

**Table 3:** Inter-operator reproducibility of densitometric and mechanical parameters.

	PE [%]		LSC [%]		ICC [-]	
	Week 18	Week 22	Week 18	Week 22	Week 18	Week 22
L	0.04	0.20	0.10	0.49	1.000	0.986
Tot BMC	0.21	0.20	0.51	0.49	0.998	0.999
Tot TMD	0.22	0.15	0.52	0.36	0.927	0.979
Tot BV	0.19	0.30	0.46	0.72	0.999	0.997
Tot TV	0.34	0.45	0.83	1.08	0.996	0.993
Tot BMD	0.29	0.31	0.70	0.74	0.992	0.992
Tot BV/TV	0.20	0.23	0.48	0.56	0.995	0.992
AvTotArea	0.32	0.31	0.78	0.74	0.992	0.993
AvBoneArea	0.18	0.15	0.43	0.35	0.998	0.999
MinTotArea	0.08	0.12	0.18	0.29	1.000	0.999
MinBoneArea	0.16	0.11	0.38	0.26	0.999	0.999
BMC in 40 sectors	0.18 – 2.34	0.25 – 4.25	0.43 – 5.61	0.59 – 10.21	0.875 – 0.999	0.608 – 0.999
Stiffness	3.94	4.09	9.46	9.81	0.826	0.814
Failure load	1.96	1.87	4.71	4.48	0.882	0.906

PE, precision error; LSC, least significant change; ICC, intraclass correlation coefficient.

The effect of PTH measured by each operator, in terms of difference between week 18 and week 22 of age, is reported in **Table 4**. The maximum difference between repetitions or operators was 2% and standard deviations were similar among operators. The effect of PTH increased the total BMC by 22-23%, the bone stiffness by 22-25%, and the bone strength by 21-22%.

**Table 4:** Variations between week 18 (pre-treatment) and week 22 (after 5 weeks of PTH injections) of age measured in different repetitions and by different operators (average ± standard deviation, N = 5).

		Difference in BMC [%]	Difference in Stiffness [%]	Difference in failure load [%]
Intra-operator (Operator1)	Repetition1	23 ± 2	24 ± 1	22 ± 1
	Repetition2	22 ± 2	23 ± 2	21 ± 1
	Repetition3	22 ± 2	25 ± 3	22 ± 2
Inter-operator	Operator1 (Rep1)	23 ± 2	24 ± 1	22 ± 1
	Operator2	22 ± 3	22 ± 2	21 ± 2
	Operator3	23 ± 3	23 ± 4	21 ± 2

**Figure 3:**
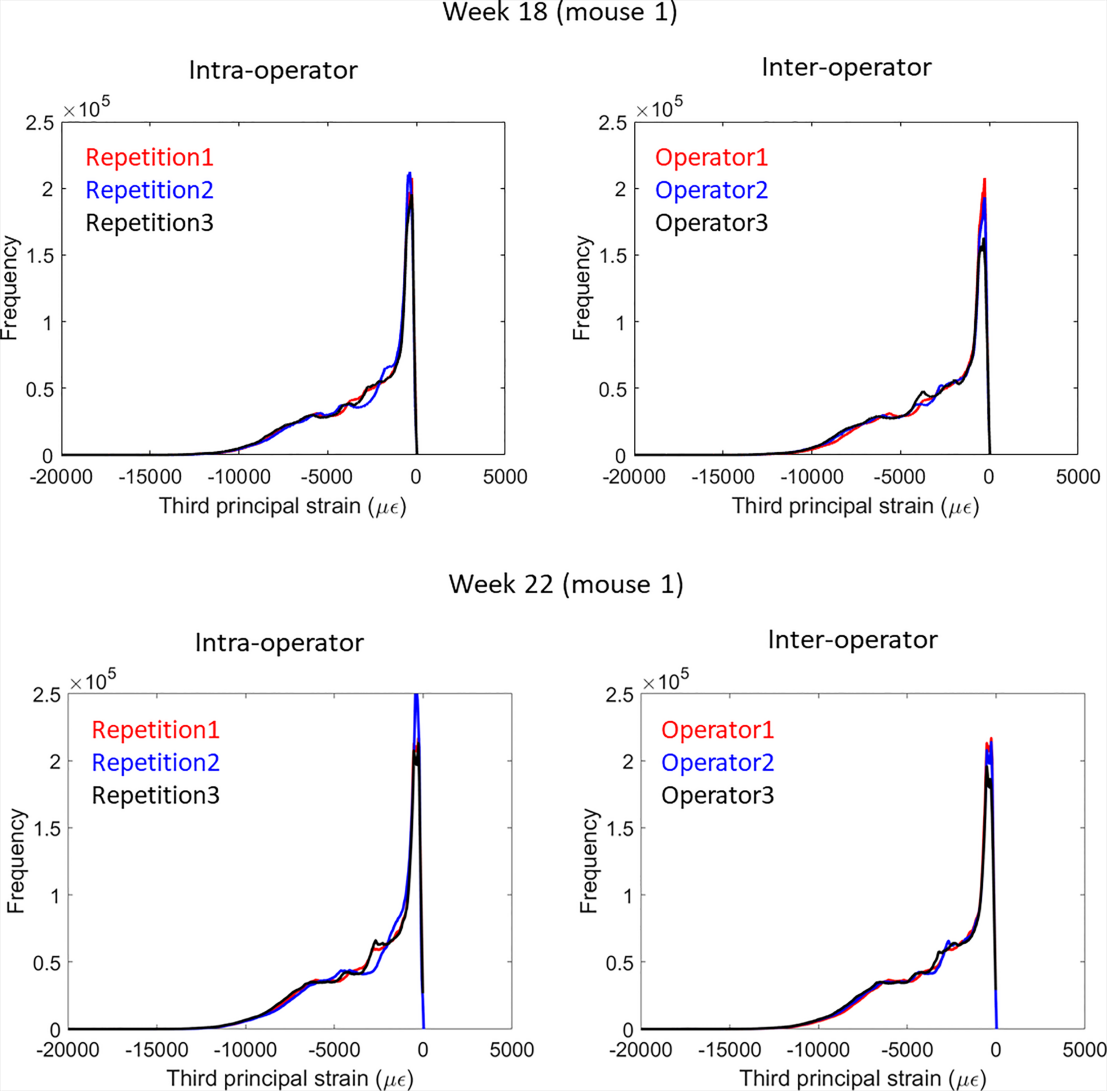
Frequency plots for the distribution of the third principal strain obtained from intra-operator and inter-operator microFE analyses of one mouse tibia at week 18 (top) or 22 (bottom) of age.

Strain distributions obtained with microFE models from repetitions by the same operator and different operators were very similar (example in **Figure 3**).

## Discussion

In this study, intra- and inter-operator reproducibility were evaluated for densitometric and mechanical properties of the mouse tibia measured from microCT images. Evaluating these properties longitudinally in mice is important to study and optimize the effect of bone interventions. Therefore, for comparing results within and among laboratories it is paramount to minimize the operator-dependency of the newly developed approaches.

As expected, the intra-operator reproducibility was higher than the inter-operator reproducibility for most densitometric and mechanical parameters. Precision errors were in both cases lower than 4.25%, which was found for BMC measured in one of the 40 partitions (section 4 at the midshaft, lateral sector) at week 22 for the inter-operator assessment. In this partition, the measured effect of PTH was 20.82%, which is approximately five times higher than the precision error (4.25%) and twice the least significant change (10.21%). Similarly, for stiffness estimated with microFE models, precision error was 4.09% and least significant change was 9.81%, while the measured difference after four weeks of treatment was 23.16%. Therefore, the method could measure the variations adequately.

Trabecular morphometric parameters were less reproducible than densitometric and mechanical properties. This is likely due to the fact that regions of interest for morphometric analyses are usually contoured manually, potentially leading to higher variability, which can be improved by an adequate level of experience of the operator (22). Additionally, trabecular parameters are more sensitive to variations in the threshold level (21) due to the presence of thin structures. In this study, reproducibility obtained for trabecular and cortical parameters were in line with that reported in literature (7, 22).

The protocol used in this study to evaluate the spatial distribution of BMC was defined with the aim to take into account that in mice growth spans across the whole animal’s lifetime, and subsequently elongation of the tibia may occur between consecutive time points in a longitudinal study (7). This elongation is taken into account by adapting the volume of interest to the tibia length and identifying corresponding subvolumes in consecutive time points by dividing the tibia into partitions. The high reproducibility of the method and ability to measure local bone changes following an intervention confirmed its applicability for *in vivo* assessments.

Mechanical properties estimated from microFE models were also adequately reproducible. Stiffness was more sensitive to the longitudinal alignment, probably due to the tibia curvature which generates a combination of compression and bending under load. Nevertheless, the registration procedure to a reference tibia was adequate to achieve a reproducible alignment and minimize the measurements variability. Probably the simplified boundary conditions contributed to the high reproducibility of the estimated mechanical properties. Conversely, in previous studies the sensitivity of the models to the longitudinal alignment was higher and dependent on the method applied to implement the boundary conditions (23, 24). The method used in this study has been validated against experimental measurements of stiffness and failure load in compression (8, 30).

It could be noted that the reproducibility of the approaches was slightly worse for week 18 than week 22 analysis (**Tables 1**–**3**). The BMC and FE analyses are based on automatic procedures, after registration and selection of VOI completed by the operator. Therefore, it is likely that variability is mainly influenced by any misalignment between repetitions. At week 22, images were aligned to the corresponding week 18 image, which had previously been registered to a reference. This additional step makes it more likely for week 22 measurements to have higher variability. Conversely, morphometric analyses are not fully automated and require manual operations by the operator (especially the contouring of the trabecular bone region), which is probably the main source of variability. While this variability is not expected to be consistently higher or lower for a specific time point, in this study the operators have started the analyses from the week 18 images and therefore may have got more used to the images and more repeatable for week 22.

A limitation of the study is that reproducibility was evaluated only for two time points of the longitudinal study (week 18, used as baseline, and week 22). However, all the steps required for image pre-processing and analysis have been included in the reproducibility assessment, in particular the alignment of the images obtained at subsequent time points to the baseline data. All other time points were analyzed using the same procedure followed in this study for the images acquired at week 22, therefore the reproducibility is expected to be similar. On the other hand, this study has remarked that the registration steps are essential to achieve reproducible measurements from microCT images acquired *in vivo*, where repeatable repositioning cannot be achieved experimentally. Another limitation is that inter-operator reproducibility for morphometric parameters was not assessed, and was assumed to be lower than the intra-operator one. Reproducibility of standard morphometric parameters has been reported previously (18, 19, 21, 22) and was analyzed here mainly for comparison with densitometric and mechanical parameters.

In conclusion, both densitometric and mechanical parameters were characterized by a high reproducibility which allows the longitudinal assessment of bone properties to evaluate the effect of interventions on the mouse tibia *in vivo*.

## Data Availability Statement

The raw data supporting the conclusions of this article will be made available by the authors, without undue reservation.

## Ethics Statement

No animal study was performed in this study but images from a previous murine study have been used. All the procedures were performed in compliance with the Animal (Scientific Procedures) Act 1986. The study was reviewed and approved by the local Research Ethics Committee of The University of Sheffield (Sheffield, UK).

## Author Contributions

Conceptualisation: SO and ED; Data curation: SO, WG, and ED; Formal analysis: SO, VC, BR, and C-AO; Funding acquisition: IB and ED; Investigation: SO, VC, BR, C-AO, and WG; Methodology: SO, VC, BR, IB, and ED; Project administration: SO and ED; Resources: IB and ED; Software: SO and WG; Supervision: IB and ED; Visualisation: SO; Writing – Original Draft Preparation: SO; Writing – Review and Editing: VC, BR, C-AO, WG, IB, and ED. All authors contributed to the article and approved the submitted version.

## Funding

The study was partially funded by the UK National Centre for the Replacement, Refinement and Reduction of Animals in Research (NC3Rs, Grant number: NC/R001073/1) and by the Engineering and Physical Sciences Research Council (EPSRC) Frontier Multisim Grant (EP/K03877X/1 and EP/S032940/1).

## Conflict of Interest

The authors declare that the research was conducted in the absence of any commercial or financial relationships that could be construed as a potential conflict of interest.

## Publisher’s Note

All claims expressed in this article are solely those of the authors and do not necessarily represent those of their affiliated organizations, or those of the publisher, the editors and the reviewers. Any product that may be evaluated in this article, or claim that may be made by its manufacturer, is not guaranteed or endorsed by the publisher.
