# Supplementary material for: Reproducibility of Densitometric and Biomechanical Assessment of the Mouse Tibia From In Vivo Micro-CT Images
Source: Front Endocrinol (Lausanne). 2022 Jun 30;13:915938. doi: 10.3389/fendo.2022.915938 (PMC9282377; doi:10.3389/fendo.2022.915938)
Supplement: Supplementary file 1 [file DataSheet_1.docx]

Supplementary Material

A schematic of the webservice developed for automatic analyses of mouse tibia microCT images is reported in Supplementary Figure 1.


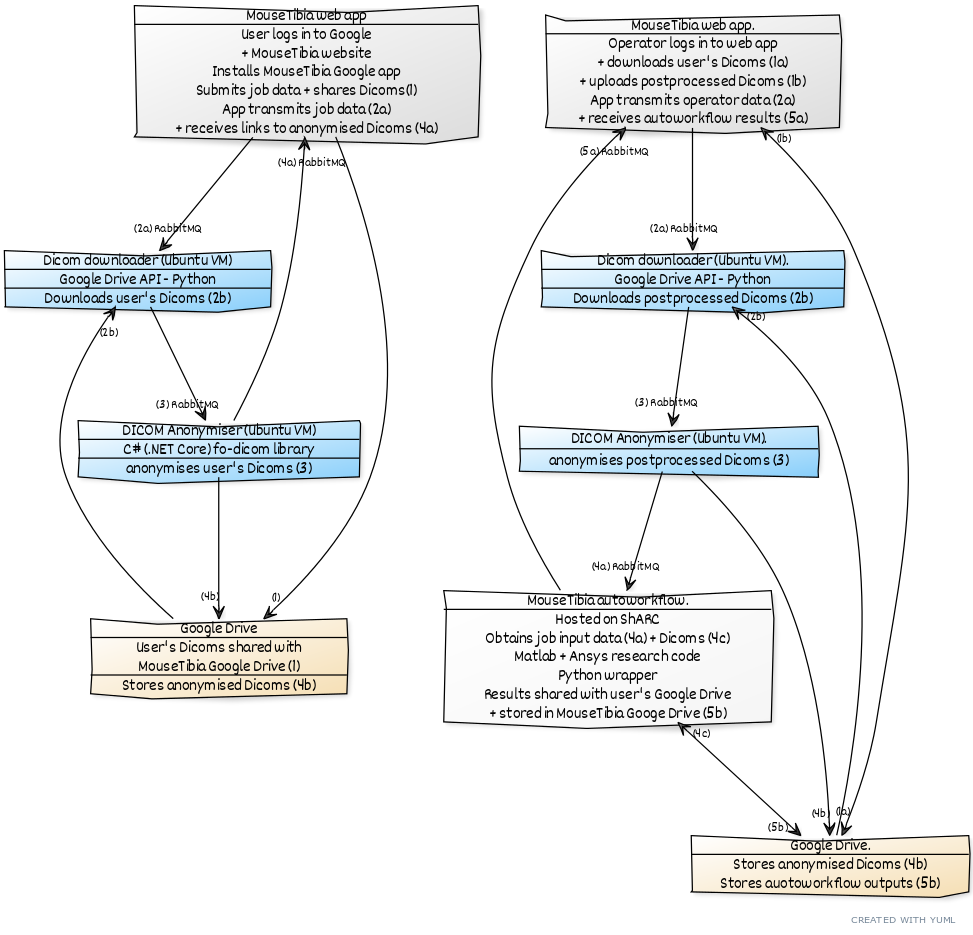


**Supplementary Figure 1**. Schematic of the MouseTibia webservice (<https://mousetibia.insigneo.org/uct2ufe/>). Two workflows are triggered by the user (left) and operator (right) respectively. The first workflow is triggered when the user submits their job data and microCT data, while the second is triggered by the operator when uploading pre-processed images.
